# Loneliness, Gender, and Relationships in Later Life

**DOI:** 10.1093/geroni/igaf122.1937

**Published:** 2025-12-31

**Authors:** Jeffrey Stokes, Heather Farmer, Katherine Fiori

## Abstract

Loneliness is a major public health concern among the older population. However, not all lonely persons are socially isolated, and increasingly, research has examined loneliness as a relational experience with implications for social partners. For instance, loneliness can be “contagious” within couples and social networks, and can generate stress, leading to adverse outcomes for intimate partners. Moreover, the experience, determinants, and implications of loneliness are gendered in later life. This symposium explores the relational causes and consequences of loneliness for older women and men. First, Stokes and colleagues discuss how verbal fluency and memory problems in an intimate partner predict spouses’ loneliness longitudinally, finding stronger effects among women and older partners. Second, Ermer and colleagues explore dyadic partners’ loneliness and friendship characteristics over time, finding gendered patterns, where women’s loneliness was associated with reduced friendship support among men four years later. Third, Rippon and colleagues focus on minoritized older couples, finding that men’s loneliness affects their partners’ life satisfaction as well as their own. Lastly, Patterson and colleagues explore the role of friendship characteristics for older adults’ sense of companionship and isolation with age. As Discussant, Dr. Fiori will contextualize these studies within the broader literature on loneliness, friendship, and relationships among the aging population, and will suggest fruitful avenues for future research. This symposium will provide new insights into the interpersonal consequences of loneliness and highlights how gender may play an important role in determining risk, which has implications for future studies and interventions seeking to reduce loneliness.

